# Dual-function ethoxylated cationic surfactant for pipeline corrosion and sulfate-reducing bacteria control: electrochemical and theoretical studies

**DOI:** 10.1038/s41598-025-30093-3

**Published:** 2025-12-14

**Authors:** N. M. El Basiony, E. A. Elsharaky, A. S. El-Tabei

**Affiliations:** Egyptian Petroleum Research Institute (EPRI), Nasr City 11727, Cairo, Egypt

**Keywords:** Cationic surfactant, EIS, XPS, DFT, Langmuir isotherm, Chemistry, Mathematics and computing

## Abstract

Effective corrosion inhibitors are essential for preventing metal degradation. In this study, a novel polyoxyethylene-based cationic surfactant (ECS) was synthesized and its structure was confirmed using various spectroscopic techniques, including FTIR and ¹H NMR. The ECS exhibits both surface-active and antibacterial properties due to the presence of quaternary ammonium, polyoxyethylene, and alkyl chain moieties, which facilitate its adsorption onto bacterial membranes and carbon steel (C-steel) surfaces. Potentiodynamic polarization (PDP) results indicated that ECS effectively suppresses both the anodic and cathodic reactions of C-steel via blocking effect. Corrosion current density (i_corr_) of C-steel dropped to 48.7µA/cm^2^ in presence of 175 ppm ECS. Electrochemical impedance spectroscopy (EIS) revealed that corrosion proceeded via a charge transfer-controlled mechanism. 175 ppm of ECS increased the charge transfer resistance (R_ct_) of C-steel to 497.81 Ω·cm^2^, compared to 38.6 Ω·cm^2^ for the blank confirming its effective adsorption. Langmuir adsorption isotherm analysis suggested that ECS is physically adsorbed on the C-steel surface, with a calculated free energy of adsorption (ΔG_ads_) of − 27.314 kJ/mol. Furthermore, the activation energy (E_a_) increased from 25.76 to 41.49 kJ/mol in the presence of ECS, indicating the formation of an energy barrier that inhibits the C-steel dissolution process. The ECS achieved a 92% reduction in C-steel degradation. Surface characterization techniques, including scanning electron microscopy (SEM), water contact angle (WCA) measurements, and X-ray photoelectron spectroscopy (XPS), confirmed the formation of a protective ECS layer that effectively shielded the carbon steel from the aggressive attack of the HCl solution. Theoretical studies, including density functional theory (DFT) and Monte Carlo (MC) simulations, were utilized to investigate the adsorption mode of ECS molecules on the C-steel surface and to elucidate how the molecular structure influenced inhibition efficiency.

## Introduction

Corrosion is the gradual degradation of a metal surface caused by chemical or electrochemical interactions with its environment, often involving the metal dissolution and the subsequent corrosion products produced on the surface, particularly in the presence of oxidants such as oxygen or sulfur^1,2^.Metallic structures are employed in various domestic and industrial applications due to their high mechanical strength and cost-effectiveness. However, corrosion poses significant security, financial, and environmental risks by considerably reducing the lifespan of metallic structures^3–7^. A recent NACE assessment quantifies the global cost of corrosion at approximately US$2.5 trillion annually, representing 3.4% of global GDP^8^. By implementing established corrosion prevention strategies, this figure could be reduced by 15–35%, yielding potential savings of US$375–875 billion^9^. Carbon steel (C-steel) was selected as the substrate due to its widespread industrial applications, favorable mechanical properties, and cost-effectiveness^10^. The corrosive medium of 1 M HCl was chosen because hydrochloric acid is commonly used in industrial processes such as acid pickling, descaling, and oil well acidizing, where carbon steel components are frequently exposed to aggressive acidic environments^11,12^. Organic compounds containing heteroatoms with lone electron pairs are among the most promising anticorrosive agents for aqueous environments. These molecules, often referred to as organic inhibitors, function primarily through adsorption onto the metal surface, impeding the corrosion process^13–15^. A primary challenge with many organic corrosion inhibitors is their limited solubility, leading to inadequate surface coverage, especially for smaller molecules. Consequently, these compounds often provide only moderate protection. Achieving an optimal balance between hydrophilicity and hydrophobicity is crucial for enhancing corrosion inhibition^16–19^. Surfactants, possessing both hydrophilic and hydrophobic moieties, exhibit a strong affinity for interfaces. Their adsorption onto metal surfaces creates a protective barrier, inhibiting corrosion. Rougher surfaces generally corrode faster due to increased wettability, which accelerates corrosion processes^20–22^. Surfactants can mitigate this by forming hydrophobic films on metal surfaces, inhibiting water diffusion and reducing corrosion. However, surfactant effectiveness depends on factors such as molecular structure and environmental conditions^23,24^.

Low cost, ease of production, and high corrosion inhibition efficiency endow cationic surfactants high potential applications^25^. These surfactants excel at reducing surface tension and forming dense interfacial layers. Literature suggests that various cationic surfactants, including quaternary ammonium, pyridine, and imidazole derivatives, offer potent corrosion protection, often at low concentrations^26,27^. However, some require higher doses for optimal performance. Given their effectiveness and versatility, polyoxyethylene-based cationic surfactants were selected for this study. These surfactants combine the advantages of oxyethylene, quaternary ammonium, and long-chain alkyl groups, enhancing adsorption to steel surfaces and creating a protective barrier against corrosion while also exhibiting antimicrobial properties against SRB^28^. Polyoxyethylene-based cationic surfactants were chosen for this study because they integrate the beneficial features of oxyethylene units, quaternary ammonium groups, and long-chain alkyl moieties, which together enhance their adsorption onto steel surfaces and facilitate the formation of a protective barrier against corrosion. Additionally, the presence of quaternary ammonium groups imparts notable antimicrobial properties, making these surfactants effective against a range of microorganisms, including sulfate-reducing bacteria (SRB). The resulting protective film significantly reduces corrosion rates by displacing aggressive ions and water from the metal interface. The combination of surface activity, corrosion inhibition, and antimicrobial efficacy underscores their suitability for applications requiring both material protection and microbial control. The prepared surfactant demonstrates exceptional corrosion inhibition by combining the advantages of both cationic and nonionic moieties. Its cationic component enables strong electrostatic adsorption on metal surfaces, forming dense hydrophobic films that effectively block corrosive agents, while the incorporated oxyethylene (nonionic) moiety enhances performance through hydrogen bonding and dipole interactions, ensuring pH stability and consistent adsorption across diverse environments. This dual functionality provides excellent surface coverage, thermal stability, and versatility without being compromised by solution pH or ionic interference. Environmentally, the surfactant’s nonionic characteristics—ethoxylated compounds—contribute to higher biodegradability and lower aquatic toxicity compared to conventional cationic inhibitors, aligning with sustainable corrosion protection strategies. The synergistic design thus balances robust inhibition with reduced ecological impact, making it suitable for industrial applications where both efficiency and environmental compliance are critical^29–32^.

In this research, we aim to synthesize an amphiphilic compound featuring dissimilar hydrophilic head groups—specifically, a combination of cationic (quaternary ammonium) and nonionic (oxyethylene) moieties—alongside a long-chain alkyl group and a bromide counterion. The integration of these structural elements is designed to significantly enhance the compound’s adsorption onto metal surfaces, as the presence of multiple or mixed head groups and long hydrophobic tails promotes strong surface activity and aggregation. Furthermore, the cationic and nonionic head group combination, together with the long alkyl chain, facilitates efficient permeation through microbial cell membranes, resulting in superior antimicrobial activity compared to conventional single nonionic or cationic surfactants. The bromide ion as a counterion can further influence adsorption properties at interfaces, supporting the formation of stable, protective layers on metal surfaces. This molecular design is expected to yield a multifunctional amphiphile with both robust corrosion inhibition and potent antimicrobial efficacy. To gain a comprehensive understanding of inhibitor adsorption behavior, computational methods like Monte Carlo (MC) simulations and density functional theory (DFT) have gained prominence in corrosion science MC simulations, in particular, provide valuable insights into inhibitor molecule orientation and adsorption on metal surfaces, help the development of high-performance corrosion inhibitors^33–35^.

This study focuses on the development of a novel polyoxyethylene-based cationic surfactant (ECS). Its chemical structure was verified using FTIR and ¹H NMR spectroscopy, while surface activity was assessed through surface tension and conductivity measurements. The ECS’s efficacy as an inhibitor for SRB and corrosion inhibitor for both C-steel in acidic environments was evaluated. Different electrochemical techniques, EIS and PDP, used to study the ECS’s activity under varying concentrations and temperatures accompanied with surface analysis (WCA, SEM, XPS). To further elucidate the inhibition mechanism, the DFT calculations, and MC simulations were conducted.

## Experiments

### Material sources

We purchased 1-bromodecane from Sigma-Aldrich (Germany), 3-(Dimethylamino) propan-1-ol from Alfa Aesar (Germany), and Ethylene oxide (EO) gas from Egyptian Gas Company. All remaining chemicals were of technical purity and utilized without further purification.

### Synthesis of N-decyl-1-hydroxy-N, N-dimethyl-3,6,9,12,15-pentaoxaoctadecan-18-aminium bromide

**Scheme 1:**
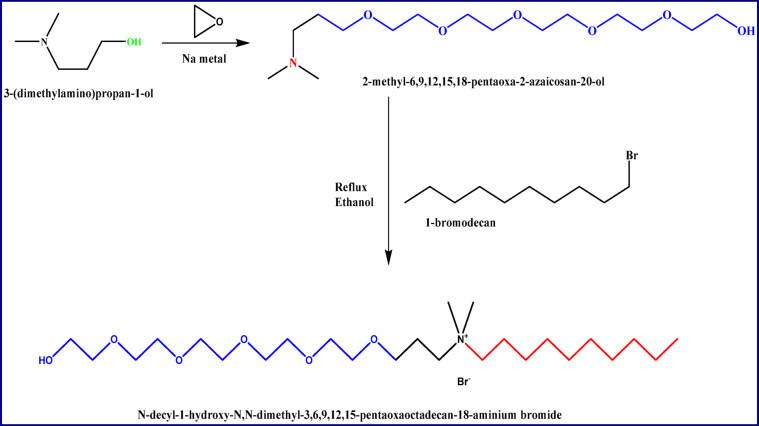
Synthesis of N-decyl-1-hydroxy-N, N-dimethyl-3,6,9,12,15-pentaoxaoctadecan-18-aminium bromide.

As illustrated by Scheme 1, a novel polyoxyethylene- quaternary ammonium surfactant (ECS) was prepared in two steps. The first step’s syntheses was demonstrated previously^23^. The second step illustrated as follows:

By refluxing 0.01 mol 2-methyl-6,9,12,15,18-pentaoxa-2-azaicosan-20-ol with 0.01 mol 1-bromodecane in ethanol for 24 h. The solution was set aside to cool. The resultant substance was purified using n- hexane and then diethyl ether to get N-decyl-1-hydroxy-N, N-dimethyl-3,6,9,12,15-pentaoxaoctadecan-18-aminium bromide as a light brown viscous liquid.

The chemical composition of the synthesized compound was verified via FTIR, and 1HNMR spectroscopy.

### Surface tension and conductivity measurements

The surface tension and conductivity of ECS solutions, prepared with double-distilled water (surface tension: 72 mN/m), were measured using a Theta optical tensiometer (Attension-Biolin Scientific Company) and a conductivity meter (Type 522; Crison Instrument, S.A.), respectively, at 25 °C.

### Biocidal activity

In accordance with ASTM D4412-84 (re-approved in 1990), ECS was tested over a concentration range of 50 to 200 ppm for 21 days to determine its biocidal effect toward SRB^36^.

### Electrochemical tests (EIS and PDP)

After immersing polished C-steel (1 cm²) working electrodes in 1 M HCl solutions, both treated and untreated with ECS, for 30 min, EIS and PDP measurements were performed using an Autolab PGSTAT-128n potentiostat/galvanostat. An Ag/AgCl (3 M KCl) reference electrode and a Pt wire counter electrode were employed in a three-electrode system. EIS was performed at AC voltage of frequency range100 kHz to 0.01 Hz and amplitude of 5 mV, while PDP was conducted within ± 0.4 V around OCP at a scan rate of 1 mV/s. All potentials were referenced to Ag/AgCl. Electrochemical parameters were exported and fitted using Nova 2.1 software. Each laboratory experiment was performed twice, and the mean values were reported.

### Quantum chemical studies (QCs)

We studied the electronic structure properties of the ECS to figure out the relation between the molecular structure electronic properties and its corrosion inhibition performance. Using the Dmol3 module implemented in Materials Studio (M.S. 6.0; Accelrys software) and based on the density function theory (DFT), the full geometry optimization parameters of ECS molecule have been calculated^37^. The Dmol3 geometry optimization process parameters were medium quality convergence tolerance and medium (10^− 5^) SCF tolerance, the functional of GGA with B.O.P accompanied with Double Numerical (DN) basis set with version file 3.5. The energy of the highest occupied and lowest unoccupied molecular orbitals (E_HO_, E_LU_) for the ECS were obtained after Dmol3 geometry optimization process and used to calculate the relevant quantum chemical parameters such as (energy of band gap ($\:{\varDelta\:E}_{ECS})$, electronegativity ($\:{{\upchi\:}}_{ECS}$) and global hardness ($\:{{\upeta\:}}_{ECS}$) according to the following Eqs^25–39^.

1 $$\:\varDelta\:{E}_{ECS}={E}_{LU}-{E}_{HO}$$

2 $$\:{\eta\:}_{ECS}=\frac{\varDelta\:{E}_{ECS}}{2}$$

3 $$\:{\chi\:}_{ECS}=\frac{-({E}_{HO}+{E}_{LU})}{2}$$

To understand the adsorption of ECS over the C-steel surface, Monte Carlo simulation (MCs) process has been run via adsorption locator module to mimic this process via calculation the adsorption energy (E _ads_) of the ECS on the most stable from of iron (Fe (1 1 0)) in gas phase and in simulated acidic solution phase. MCs annealing process processed via several steps till get the most energy table form of ECS/Fe (1 1 0) system in different phases. The annealing process was run in a simulation box (37.23 A^◦^x37.23 A^◦^x 37.23 A^◦^) where the cleaved iron Fe (1 1 0) made to (15 × 15) supercell and 15 A^◦^ vacuum slap. Forcefield COMPASS (version 2.8) controlled the MCs as reported previously^38,40^.

### Surface analysis

#### Water contact angle (WCA)

WCAs were measured on polished C-steel, blank (1 M HCl), and 175 ppm ECS-treated C-steel surfaces after 12 h of immersion using a Theta optical tensiometer from Biolin Scientific.

#### SEM and XPS

After 12 h immersion in 1 M HCl alone or mixed with 175 ppm ECS, the morphology of C-steel sheets was characterized by field emission scanning electron microscopy (FESEM, JSM-IT800, JEOL, Japan), Surface chemical composition of C-steel immersed in 1 M HCl + 175 ppm ECS analyzed through X-ray photoelectron spectroscopy (XPS, Thermo Fisher Scientific, NEXSA G2, UVP, Inc., UK).

## Results and discussion

### Chemical structure verification

The characterization of 2-methyl-6,9,12,15,18-pentaoxa-2-azaicosan-20-ol has previously been explored in the published literature^23^.

#### N-decyl-1-hydroxy-N, N-dimethyl-3,6,9,12,15-pentaoxaoctadecan-18-aminium bromide

**Fig. 1:**
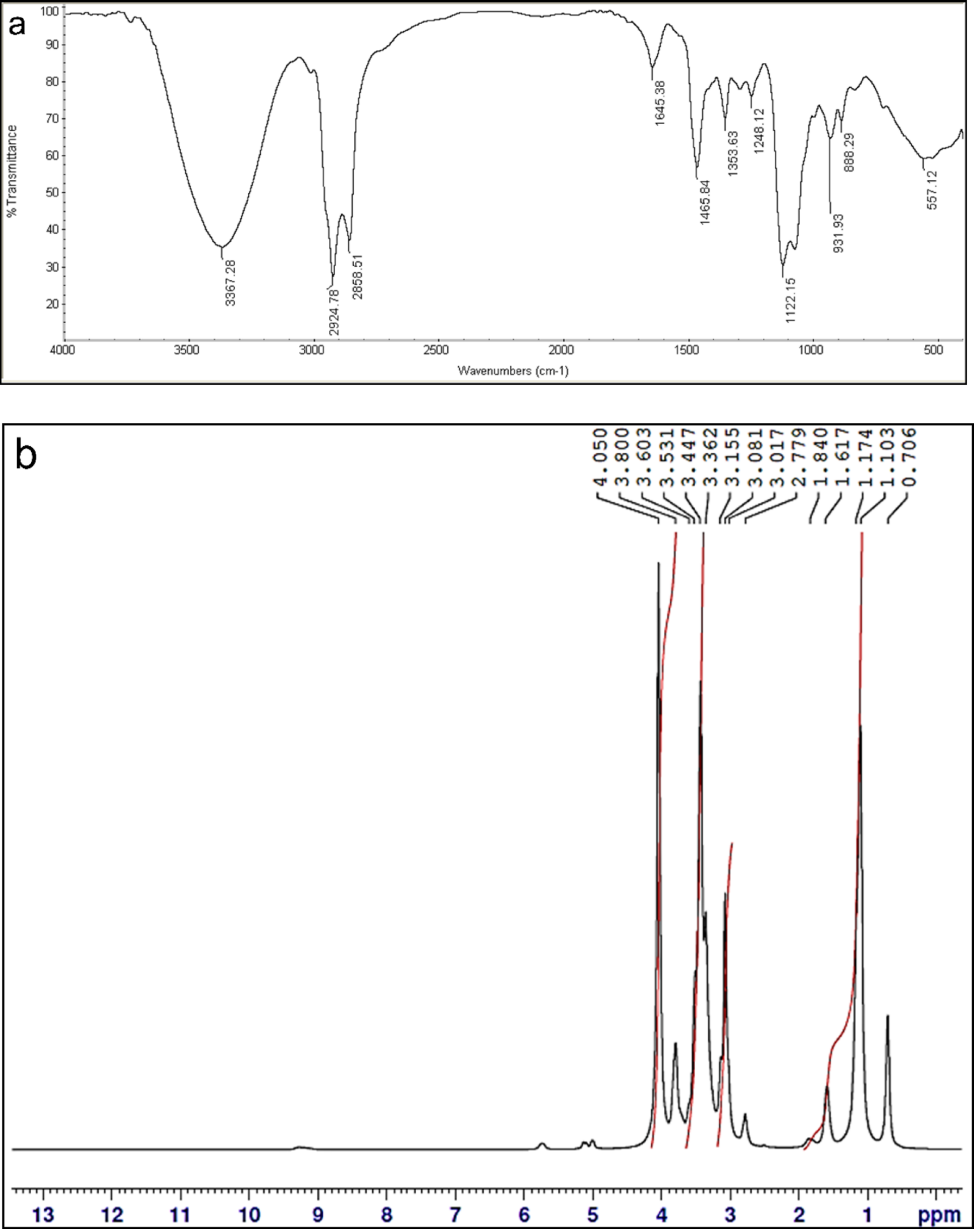
FT-IR and ^1^H NMR of N-decyl-1-hydroxy-N, N-dimethyl-3,6,9,12,15-pentaoxaoctadecan-18-aminium bromide.

FT-IR spectrum (Fig. 1a) of N-decyl-1-hydroxy-N, N-dimethyl-3,6,9,12,15-pentaoxaoctadecan-18-aminium bromide, displayed bands (cm^− 1^) at 2924.7 & 2858.5 (aliph. C-H asym. & sym. stretching); indicates the presence of long chain hydrocarbon; 1465.8 (aliph. C-H asym.bending),1122.1 (ethereal band; C-O-C asym. stretching) & 3367.7 (OH stretch**)**.

^1^HNMR (DMSO - d6) spectrum (Fig. 1b) exhibited peaks at δ: 0.70 ppm (3 H, C**H**_**3**_), δ: 1.17 ppm (16 H, (C**H**_**2**_)_8_), 2.7 ppm (4 H, **H**_**2**_CN^+^C**H**_**2**_)_8_), δ: 3.08 ppm (3 H, N^+^C**H**_**3**_), δ: 1.6 ppm (2 H, C**H**_**2**_CH_2_N^+^CH_3_), δ: 3.3–3.6 ppm (4 H, OC**H**_**2**_C**H**_**2**_O), δ: 3.8 ppm (2 H, C**H**_**2**_OH) and δ: 4.05 ppm (1H, CH_2_O**H**).

### Surface active properties

**Fig. 2:**
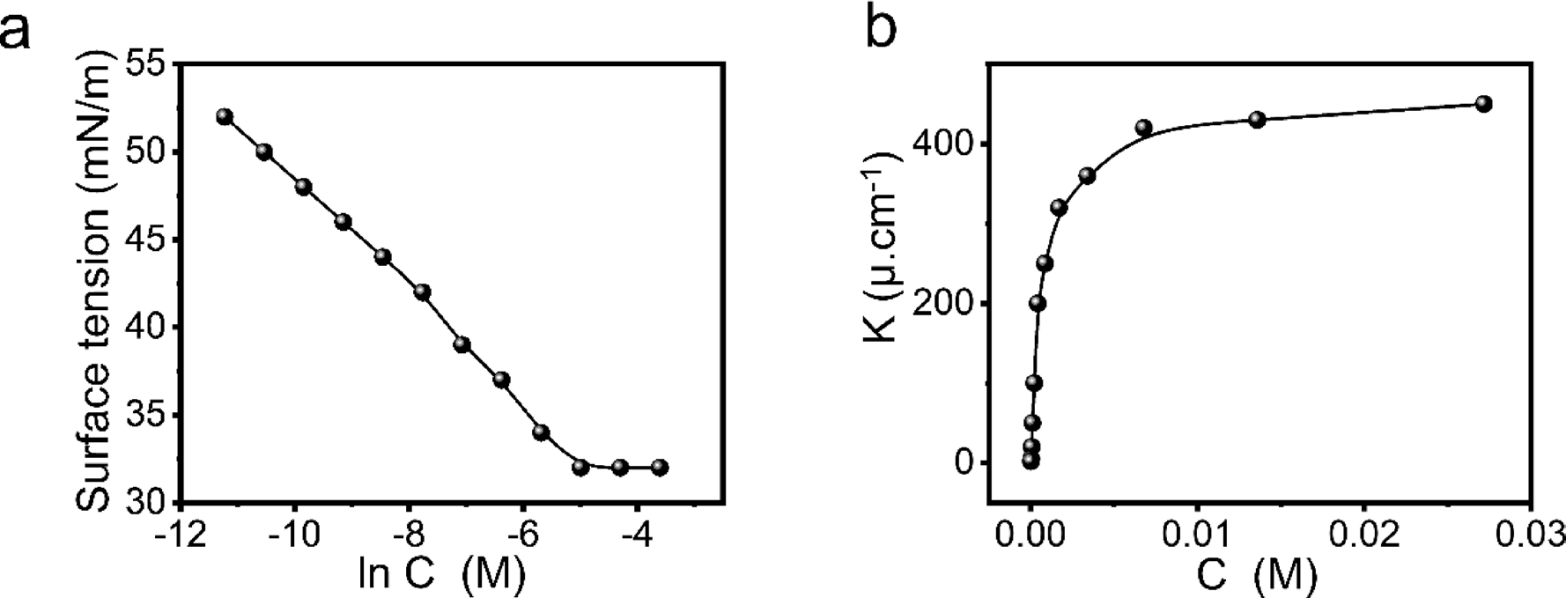
**a**) Variation of the surface tension with concentrations of ECS, **b**) plotting of electrical conductivity against ECS concentrations in double distilled water at 25 °C.

The prepared inhibitor has good solubility in polar solvents, such as water, methanol, ethanol, and acetone. Also, surface tension measurements supported with their corresponding parameters in Table 1; Fig. 2 were applied in water and reflected high solubility of the prepared surfactant with a CMC value of 0.0067 **mol L**^**− 1**^.

Figure 2a depicts the variance in surface tension values of ECS solutions. Surface tension values decline sharply as concentration increases, then the curve breaks quite quickly at still relatively low concentrations, eventually reaching a stable state as concentrations rise^41^. The critical micelle concentration (CMC) was calculated by intersecting the *γ-*ln *C* curve and listed in Table 1. The obtained CMC value for the synthesized surfactant is 0.0067 mol L^− 1^.

Surface tension data (γ) at CMC were utilized to calculate surface pressure (effectiveness) value from equation^42^:

4 $$\:{\pi\:}_{\text{c}\text{m}\text{c}}={\gamma\:}_{\text{o}}-{\gamma\:}_{\text{c}\text{m}\text{c}}$$

where$\:\:{\gamma\:}_{\text{o}}$ denotes the surface tension of fresh water at the suitable temperature and $\:{\gamma\:}_{\text{c}\text{m}\text{c}}$ denotes the surface tension at CMC. Table 1 shows the$\:\:{\pi\:}_{\text{c}\text{m}\text{c}}$ value of the prepared ECS. It noticed that the$\:\:{\pi\:}_{\text{c}\text{m}\text{c}}$ has a high value may be due to the presence of quaternary nitrogen, oxyethylene chain and 10-C aliphatic chain moieties in the ECS which enhance the adsorption affinity at the interface and hence decrease γ value.

The molecular structure of ECS also affected the surface excess (*Γ*_max_) which was calculated according to Gibb’s equation^42^:

5 $$\:{\varGamma\:}_{\text{m}\text{a}\text{x}}=\left(\frac{-1}{nRT}\right)\left(\frac{d\gamma\:}{d\text{ln}C}\right)\:\:$$

Where, R is the universal gas constant, T is the absolute temperature and the value of n is number of species ions in solution. The *Γ*_max_ value was calculated and listed in Table 1.

The area occupied by each molecule at the liquid/air interface in nm^2^ is known as the minimum surface area ($\:{A}_{\text{m}\text{i}\text{n}}$). The following equation is used to calculate $\:{A}_{\text{m}\text{i}\text{n}}$^43^:

6 $$\:{A}_{\text{m}\text{i}\text{n}}=\frac{{10}^{14}}{{N}_{\text{A}}{\varGamma\:}_{\text{m}\text{a}\text{x}}}$$

Where, *N* is Avogadro’s number. According to the findings in Table 1, $\:{A}_{\text{m}\text{i}\text{n}}$ of the ECS is a high value. This suggests that ECS has a higher affinity for occupying a larger surface area of the substrate, thereby enhancing its adsorption on surfaces such as cell membranes or C-steel.

### Conductivity measurements

At 25 °C, conductivity measurement was taken for cationic surfactants in order to determine the CMC and the degree of counter ion dissociation (β). Specific conductivity increases linearly with surfactant concentration until the critical micelle concentration (CMC) is reached, after which the rate of increase slows **(**Fig. 2b**)**. The CMC is determined by the intersection of these linear trends, and the *β* is calculated from their slope ratio. The values of CMC and β in surfactant micelles were 0.007 mol L^− 1^, 0.015 as listed in Table 1. The CMC value, as measured by electrical conductivity, agreed with the value calculated using surface tension. The charged pseudo-phase model of micelle production states that the standard free energy (Δ*G*_mic_) of micelle production was computed by the following equation^44^:

7 $$\:\varDelta\:G_{mic}=\:(2-\beta)\:RT\:ln\:CMC$$

The ΔG_mic_ of ECS was negative, showing that micellization occurs spontaneously. The change in free energy of adsorption ($\:{\varDelta\:G}_{\text{a}\text{d}\text{s}}^{^\circ\:}$) for the synthesized surfactant was calculated and listed in Table 1 according to the following equations^27^:

8 $$\:{\varDelta\:G}_{ads}^{^\circ\:}={\varDelta\:G}_{mic}^{^\circ\:}-(0.06023\:\times\:\:{\pi\:}_{CMC}{A}_{min})$$

From the data in Table 1, the negative value of $\:{\varDelta\:G}_{\text{a}\text{d}\text{s}}^{^\circ\:}$ indicates that the adsorption process is spontaneous. Furthermore, $\:{\varDelta\:G}_{\text{a}\text{d}\text{s}}^{^\circ\:}\:$is more negative than $\:{\varDelta\:G}_{mic}^{o}$, surfactant molecule tending to adsorb at the air/water interface until total surface coverage is achieved, and then micelles form.

**Table 1:** Surface active parameters of ECS in double distilled water at 25^°^C.

Surface tension measurements					Conductivity measurements			
**CMC** **(mol L** ^**− 1**^ **)**	**γ**_**CMC**_ **(mNm** ^**− 1**^ **)**	$\:\pi\:$_**CMC**_ **(mNm** ^**− 1**^ **)**	**Г**_**max**_ **x10**^**11**^ **(mol cm** ^**− 2**^ **)**	**A**_**min**_ **(nm** ^**2**^ **)**	**CMC** **(mol L** ^**− 1**^ **)**	**β**	**ΔG**_**mic**_ **(kJ mol** ^**− 1)**^	**ΔG**_**ads**_ **(kJ mol** ^**− 1**^ **)**
0.0067	32	40	6.4	2.57	0.007	0.015	−24.40	−30.59

## Antimicrobial action

In the process known as “microbiology-influenced corrosion, (MIC) " the metabolic activity of microorganisms speeds up the corrosion kinetic rate of a metallic material^45,46^. One of the MIC species, sulfate reducing bacteria (SRB), was discovered to have the largest colony in crude oil. SRB’s role in various corrosion reactions under anoxic/anaerobic conditions has been extensively documented^27^. Petroleum’s hydrocarbons could act as electron donors for SRB, which uses sulfate as the terminal electron acceptor in respiration, resulting in the production of sulfide. Additionally, H_2_S production results in the acidification and plugging of petroleum reservoirs, as well as bio-corrosion of pipelines, and tanks’ metal surfaces^47^. As a result, current research has focused primarily on the development of biocides to combat these challenges. The results in Table 2 display that ECS has an antimicrobial effect toward SRB. Because of its unique chemical structure, which contains quaternary ammonium and oxyethylene groups, ECS has an adsorption affinity for and can permeate the microbial cellular membrane. The selective permeability of the cell was compromised which had an adverse effect on the biological reactions of the cell and ultimately resulted in cell death^48^.

**Table 2:** Antimicrobial action of the ECS against sulfate reducing bacteria (SRB).

Dose (ppm)	50	100	150	200
**Result** (Bacterial Cell/mL)	10^5^	10^4^	10^4^	10^3^

*The blank was 10^6^ cell/mL.

## Electrochemical corrosion evaluation

### Electrochemical impedance spectroscopy (EIS)

**Fig. 3:**
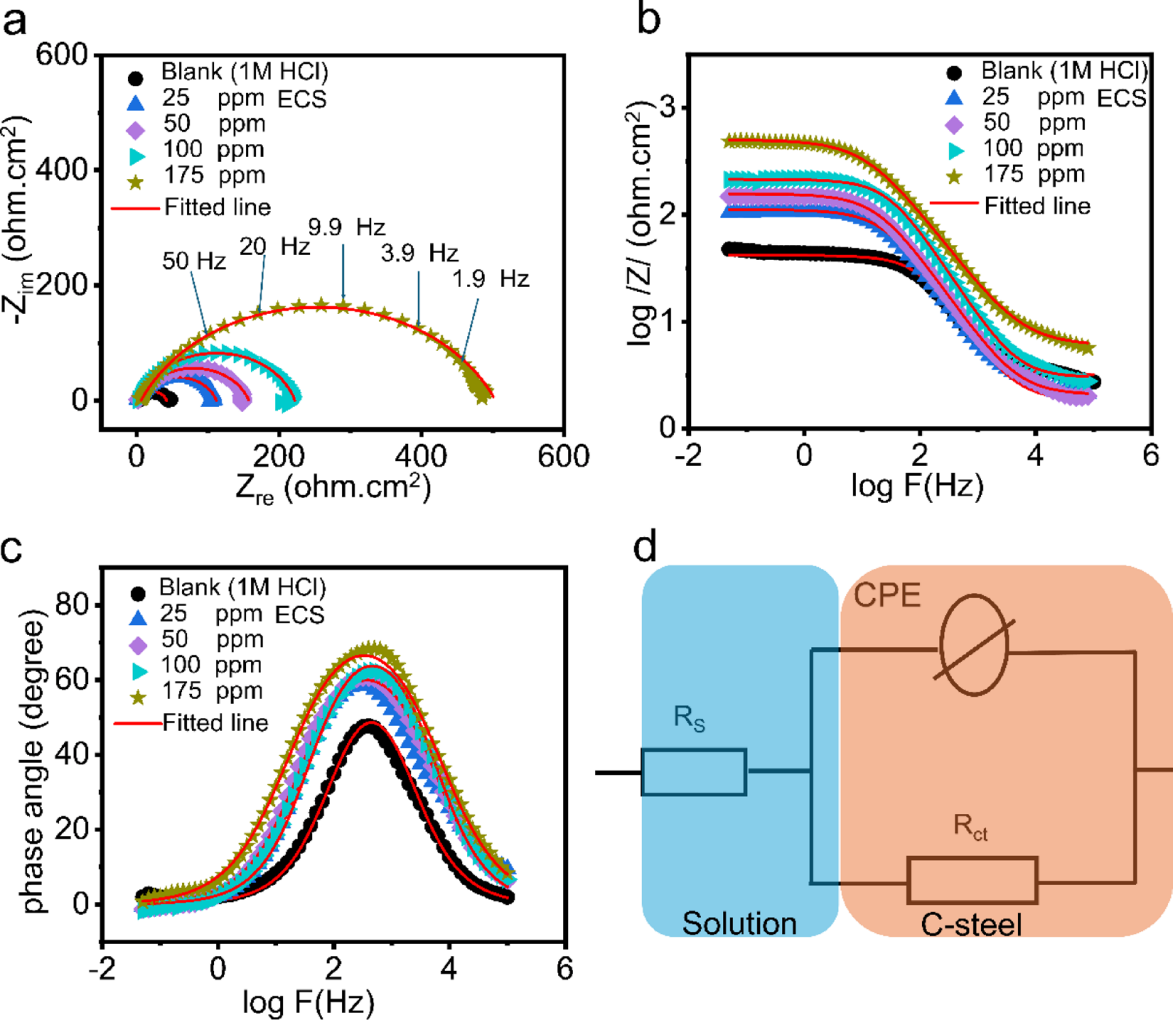
Nyquist (**a**), bode (**b**) and phase degree (**c**) diagrams for CS in 1 M HCl in absence and presence of different concentrations of ECS at 25 $^\circ C$◦C fitted with the proposed equivalent circuits (**d**).

EIS measurements at room temperature were employed to evaluate the ECS’s influence on C-steel corrosion in 1 M HCl. The similar Nyquist, Bode, and phase angle plot profiles (Fig. 3a-c) for C-steel treated and untreated with ECS indicate that the corrosion mechanism, controlled by charge transfer, remains unaffected by ECS addition^49^. As the concentration of ECS increases, the diameter of the Nyquist semicircles (Fig. 3a) significantly expands. This indicates improved anti-corrosion resistance for the carbon steel, which is due to the enhanced adsorption of ECS on its surface. The Bode modulus plots (Fig. 3b) support this observation. In the low-frequency region (log f from − 2 to −1.3), the impedance modulus (log ∣Z∣) increases as the ECS concentration rises. Furthermore, the phase angle shifts toward-90º (Fig. 3c) at intermediate frequencies. This change reflects improved capacitive behavior and confirms that ECS forms a protective barrier on the carbon steel. This barrier reduces the effective surface area available for charge exchanges, thus demonstrating a superior protective effect against the HCl solution^50^. The Nyquist curves (Fig. 3a) exhibit a depressed capacitive loop due to frequency dispersion, likely caused by the C-steel surface roughness and non-homogeneity^51^. EIS kinetics parameters for the C-steel reaction were exported by applying equivalent circuit presented in Fig. 3d and reported in Table 3. This equivalent circuit comprises solution resistance (R_s_), charge transfer resistance (R_ct_) and a constant phase element (CPE) which replaces the double layer capacitance (C_dl_) to compensate the surface heterogeneity and to get better fitted data. The CPC can be defined by the expression^52^:

9 $$\:{Z}_{CPE}=\:{Y}_{o}^{-1}.{\left(i\omega\:\right)}^{-n}$$

where, $\:{Y}^{^\circ\:}$ is a proportional factor, (); is angular frequency, i; is the imaginary unit (−1) and n is a CPE exponent and can be taken as a gauge of the surface roughness and heterogeneity. Table 3 reveals a significant increase in the R_ct_ values for C-steel upon ECS addition. The R_ct_ rises from 38.6 ohm.cm^2^ to 497.81 ohm.cm^2^ – a tenfold increase. This suggests enhanced ECS adsorption, creating a barrier that hinders direct contact between C-steel’s active sites and HCl^52^.

The inhibition efficacy (IE_z_%) of ECS was calculated based on the R_ct_ values according to the equation^53^:

10 $$\:{IE}_{Z}\%=\frac{{R}_{ct}-\:{R}_{ct\:ECS}}{{R}_{ct\:ECS}}\:x\:100$$

ECS significantly enhances C-steel surface coverage, increasing from 0.64 to 0.92 as the dosage rises from 25 to 175 ppm. This in turn increases the inhibition efficiency from 64% to 92% at the same doses. The observed decrease in $\:{Y}^{^\circ\:}$ values is attributed to the displacement of water molecules, chloride, and hydronium ions at the C-steel/HCl interface by ECS molecules, leading to a reduced capacitance of the dielectric layer and/or increasing the adsorbed ECS layer thickness according to the Helmholtz model concept^53^. Higher ECS concentrations result in a thicker adsorption layer on the C-steel surface, consequently improving inhibition efficiency^54^. The decrease in n values compared to the blank indicates increased ECS molecule accumulation on the C-steel surface^26,55,56^. The effectiveness of ECS as a corrosion inhibitor stems from its dual-action structure. The electron-rich heteroatoms (oxygen and nitrogen) are the initial points of contact, forming strong chemical bonds with the iron atoms on the steel surface. This anchors the molecule securely. Simultaneously, the long, hydrophobic 10-carbon aliphatic chain creates a physical barrier that prevents aggressive ions, such as chloride and hydronium, from reaching and attacking the C-steel’s active sites. This combined chemical bonding and physical shielding mechanism results in a highly effective protective layer that significantly inhibits corrosion^57,58^.

**Table 3:** Electrochemical impedance spectroscopy parameters for C-steel in 1 M HCl in absence and presence of different concentrations of ECS at 25$^\circ C$.

Inh.	ECS Conc. mM	ECS Conc. ppm	*R*_s_, (Ω.cm^2^)	*R*_ct_, (Ω.cm^2^)	CPE		Ɵ	IE%	X^2^
					Y° e-5 s^*n*^ Ω ^−1^ cm^− 2^	*n*			
**1 M HCl**	00	0.00	2.26	38.6	15.4	0.8565	-----	----	3.63
**ECS**	4.5 × 10^− 8^	25	2.28	107.66	11.5	0.79529	0.6414	64.14	3.42
	9.14 × 10^− 8^	50	2.29	155.35	10.3	0.7999	0.7493	74.93	2.079
	1.83 × 10^− 7^	100	2.83	220.76	7.2	0.79032	0.8253	82.53	2.079
	3.20 × 10^− 7^	175	3.98	497.81	6.6	0.7374	0.9223	92.23	1.248

### Adsorption isotherm

**Fig. 4:**
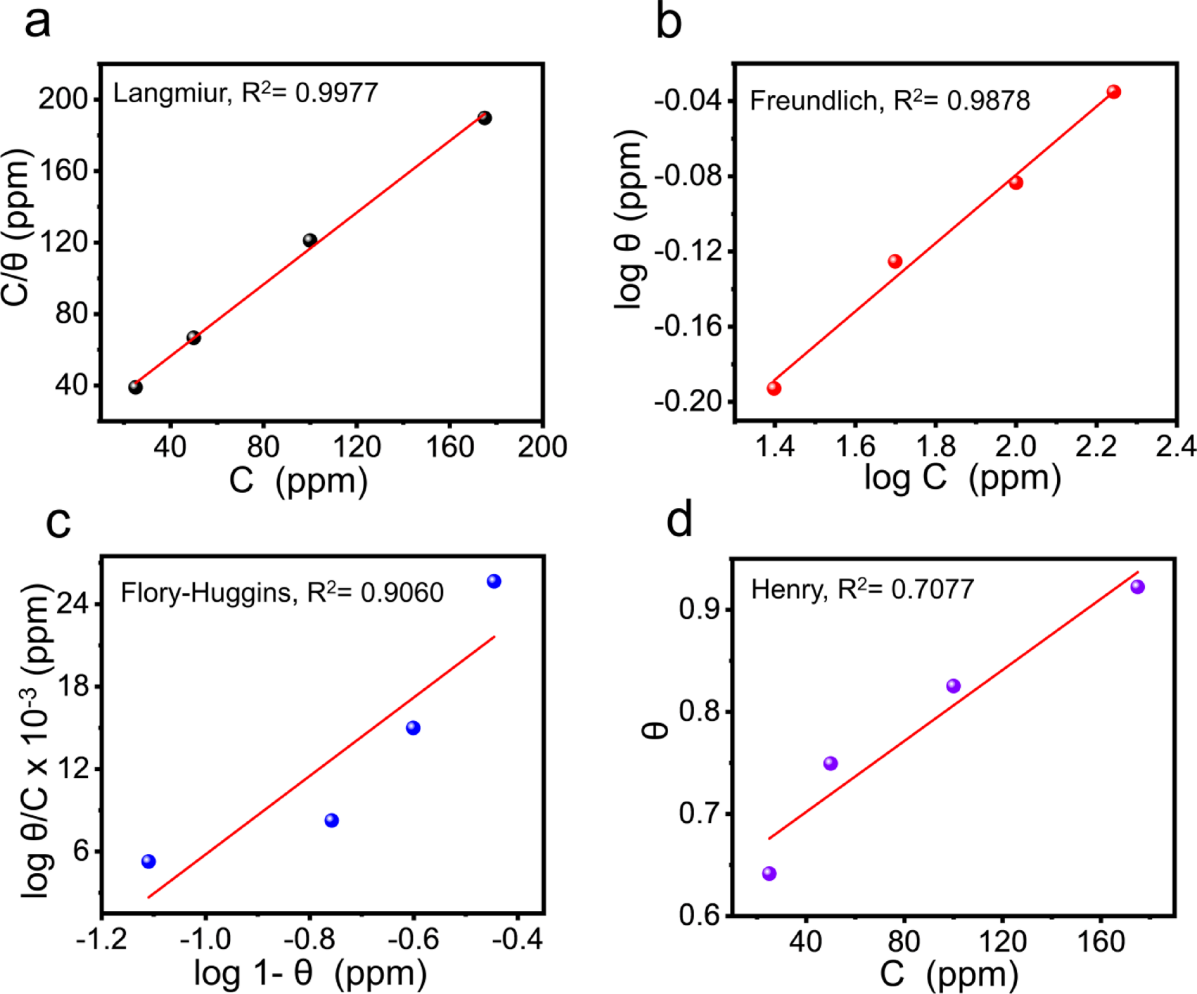
Langmuir, Freundlich, Flory-Huggins and henry adsorption isotherm models of ECS adsorption at CS/HCl interface based on EIS data at 20 ^◦^C.

Understanding the adsorption behavior of ECS at the C-steel/HCl interface is essential to elucidating their mechanisms of action. For this goal, different adsorption isotherm models are applied, Langmuir, Freundlich, Florry-Huggins and Hennry (Fig. 4), based on EIS data according to the following Eqs^59,60^. :

11 $$\:{\text{K}}_{ads}\:{C}_{ECS}={\uptheta\:}/(1-{C}_{ECS})$$

12 $$\:log\theta\:=log{K}_{ads}+\frac{1}{n}log{C}_{ECS}$$

13 $$\:log\frac{\theta\:}{{C}_{ECS}}=log{K}_{ads}+xlog(1-\theta\:)$$

14 $$\:{\text{K}}_{ads}\:{C}_{ECS}={\uptheta\:}$$

where,$\:\:({K}_{ads}$) is the adsorption equilibrium constant, (1/n) is the Freundlich constant, (χ) Flory-Huggin constant.

The best-fitting isotherm model was determined based on the correlation coefficient (R²) value. A higher R² indicates a better fit. According to the data in Table 4, among the various isotherm models applied, the Langmuir adsorption isotherm most accurately represents the adsorption of ECS on the C-steel surface (Fig. 4a). The monolayer of ECS adsorbed on the C-steel surface, according to the Langmuir isotherm, enhances both physical and chemical interactions. This protective layer occupies the C-steel’s active sites, hindering direct contact with HCl^61^. The standard free energy change (ΔG_ads_) of C-steel in presence of ECS at room temperature is function of K_ads_ according to the Eqs^61,62^. :

15 $$\:{-\varDelta\:G}_{ads}=2.303RTlog\left({10}^{6}x{K}_{ads}\right)$$

where, (10^6^) is the water concentration in ppm unit and (R) is the standard gas constant.

The adsorption of ECS on C-steel was well described by the Langmuir isotherm, which assumes monolayer adsorption on a uniform surface with a fixed number of identical sites and negligible lateral interactions between adsorbed molecules. In the present case, the calculated ΔG_ads_ value (−27.314 kJ mol^− 1^) indicates that the adsorption process is predominantly physisorption, driven by electrostatic attraction between the positively charged quaternary nitrogen/terminal protonated hydroxyl groups of ECS and the negatively charged steel surface (due to pre-adsorbed Cl^−^ ions). However, the molecular structure of ECS also allows chemisorption contributions via electron pair donation from oxygen atoms (in the ethylene oxide units) to vacant Fe 3 d orbitals. This mixed physisorption–chemisorption behavior can still follow Langmuir characteristics when the adsorbed layer is compact, the interaction energies are uniform, and each site is occupied by one inhibitor molecule. Therefore, the Langmuir model provides a good approximation for describing the dominant adsorption mode, even though a secondary chemisorption pathway is also present^63,64^.

**Table 4:** Output isotherm parameters for the adsorption of the prepared ECS at CS/HCl surface interface using different models at 25 ^o^ C.

Isotherm model for ECS adsorption	Parameters		*R* ^2^	-ΔG_ads_ (kJmol-1)
Langmuir	Slope	1.0041	0.9977	27.314
Freundlich	1/n	0.18161	0.9878	31.705
Flowry-Huggins	x	0.02853	0.9060	34.427
Henry			0.7077	18.486

### Potentiodynamic polarization measurements (PDP)

**Fig. 5:**
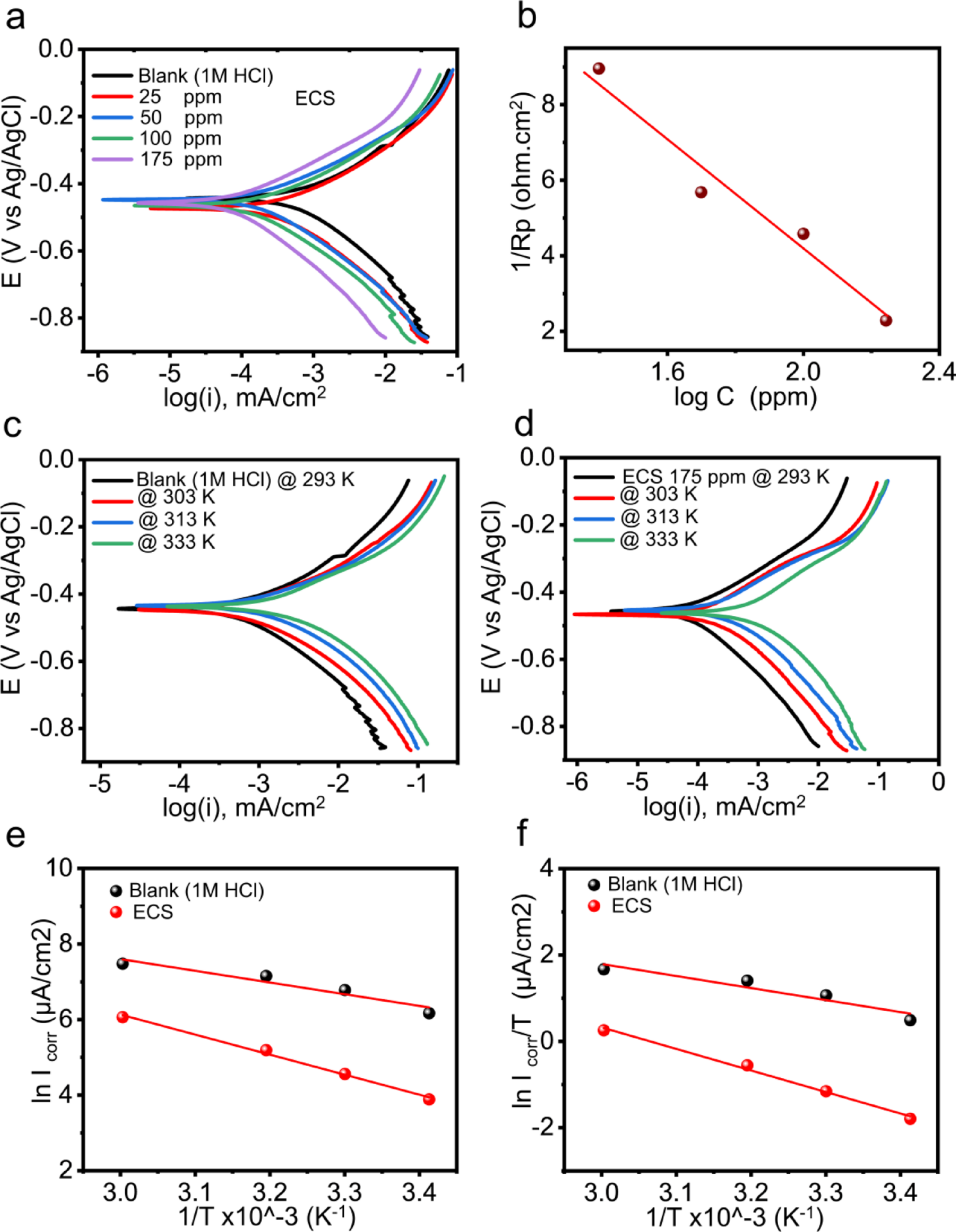
(**a**) Potentiodynamic polarization curves for C-steel in 1 M HCl in absence and presence of different concentrations of ECS at 25 ^◦^C. (**b**) Variation of the reciprocal of polarization resistance (1/Rp) versus the logarithm concentration of the ECS, log C_inh_, of the C-steel in 1 M HCl. (**c**,** d**) the Potentiodynamic polarization curves for C-steel at different temperatures in absence and presence 175 ppm of ECS, respectively. (**e**) Arrhenius plots, variation of ln i_corr_ against 1/T, for C-steel in 1 M HCl devoid of and containing 175 ppm of ECS and (f) Transition state relation of ln (i_corr_/T) against 1/T, for C-steel in 1 M HCl devoid of and containing 175 ppm of ECS.

Figure 5a displays the PDP response of C-steel immersed in pure 1 M HCl and after the ECS addition in different concentrations (25–175 ppm) under static conditions at room temperature. The similar shape of i-v curves of C-steel indicates the ECS has no effect on the C-steel dissolution mechanism^65^. ECS shifts the C-steel polarization curves towards the noble region, indicating its corrosion inhibition properties^66^. The presence of active centers, such as quaternary nitrogen and protonated terminal hydroxyl groups, in the ECS molecule promotes its adsorption and favorable orientation on the metal surface through electrostatic attraction. Additionally, electron donor–acceptor interactions between the lone pairs of oxygen atoms in the ethylene oxide groups and the vacant 3 d orbitals of iron, combined with the hydrophobic effect of the alkyl chain, facilitate the formation of a compact and robust adsorption layer. This synergistic interaction underlies the high inhibition efficiency of ECS^62^. Kinetic parameters for the C-steel corrosion reaction were determined by extrapolating the i-v curves in the Tafel regions. Corrosion potential (E_corr_), current density (i_corr_), and Tafel slopes (β_a_ and β_c_), were used to evaluate the inhibition performance of ECS and elucidate the reaction mechanism at the C-steel/HCl interface. The results are summarized in Table 5. The insignificant change in the corrosion potential (E_corr_), lower than 85 mV, of C-steel after the addition of ECS suggests that ECS has a blocking effect on both the anodic and cathodic sites of C-steel, indicating that ECS acts as a mixed-type inhibitor^64^. The observed decrease in Tafel slopes to a constant value after ECS addition implies that, while the overall corrosion mechanism remains unchanged, the inhibitor may be influencing the rate-determining steps of the anodic and cathodic reactions. This could result in changes in the Tafel slopes without altering the overall corrosion process reaction pathway, consistent with the observations from EIS measurements. Additionally, the parallel appearance of the cathodic curves suggests that hydrogen ion reduction at the C-steel surface ($\:2{H}^{+}+2{e}^{-}\:\to\:{H}_{2\uparrow\:})$ primarily occurs via a charge-transfer mechanism^66^. ECS exhibits a more pronounced inhibitory effect on the cathodic reaction compared to the anodic reaction, as evidenced by the higher absolute value of βc relative to βa^67–69^(Table 5). The alkyl chain of ECS endows significant hydrophobicity (as discussed in Sect. 6.1), inhibiting the migration of water and chloride ions and enhancing the resistance of the C-steel passivation film. Based on the values of C-steel corrosion current density in absence (i_corr_) and after ECS addition in different doses (i_ECS_), surface coverage (θ) and inhibition efficiency percentage (η_ECS_ %) can be calculated according to the Eqs^68,70^. :

16 $$\:\theta\:=({i}_{corr}-{i}_{ECS}/{i}_{corr}\:)$$

17 $$\:{\eta\:}_{ECS}\%=\:\theta\:\times\:100$$

Table 5 shows a significant decrease in i_corr_ from 477.56 µA/cm² for the blank to 48.72 µA/cm² with ECS addition. Inhibition efficiency increases to approximately 90% at 175 ppm ECS concentration. Moreover, polarization resistance (R_p_) of C-steel, is an electrochemical parameter that represents the slope of the potential–current density curve near the corrosion potential (E_corr_). Physically, R_p_ is a measure of how strongly the metal surface resists the passage of corrosion current when a small perturbation in potential is applied, is function of Tafel slope and i_corr_ according to the Stern-Geary equation^71^:

18 $$\:{R}_{p}=\:\frac{Y}{{I}_{corr}}$$

Where, Y is constant and can be calculated as: $\:Y=\frac{{\beta\:}_{a}{\beta\:}_{c}}{2.303({\beta\:}_{a}+{\beta\:}_{c})}$. From this equation, an increase in R_p_ reflects either a decrease in i_corr_ or a change in reaction kinetics (Tafel slopes), both of which indicate improved corrosion resistance. According to Stern-Geary equation, an increase in R_p_ can arise from a decrease in i_corr_. In the presence of ECS, adsorption of its molecules on the C-steel surface blocks active sites and limits the accessibility of corrosive species (Cl^⁻^, H^⁺^) to the metal. This barrier effect slows anodic metal dissolution and cathodic hydrogen evolution.

Figure 5b, display the straight-line relationship between $\:{R}_{p}^{-1}$ and logarithm ECS concentration according to the Eqs^71,72^. :

19 $$\:{{R}_{p}^{-1}={a}_{1}-{b}_{1}logC}_{ECS}$$

where, a_1_ and b_1_ are constants^73,74^.

The increase in ECS concentration leads to a marked rise in R_p_ ​, indicating enhanced resistance to charge transfer at the C-steel surface. This improvement arises from the adsorption of ECS molecules, which block active corrosion sites and form a protective barrier, thereby reducing the corrosion rate. The polarization (PDP) results are consistent with the EIS findings, confirming the effective inhibition mechanism.

### Effect of temperature

The influence of temperature on C-steel corrosion reaction was studied with and without 175 ppm ECS addition in the 303–333 K range (Fig. 5c **and d**). As expected, higher temperatures accelerated corrosion rate, shifting polarization curves towards the active region and increasing i_corr_ values (Table 6). Elevated temperatures boost electrolyte conductivity and kinetic energy, facilitating ions transport and speeding up corrosion rate. Hydrogen gas evolution at higher temperatures disrupts the protective layer on C-steel, leading to an increase in the exposed metal surface area^75^. A 14% reduction in ECS inhibition efficiency was observed with a threefold temperature increase, highlighting its potential as a corrosion inhibitor for C-steel in 1 M HCl^76,77^.

Based on the values of i_corr_, listed in Table 6, in presence and absence of 175 ppm ECS at different temperatures, thermodynamic activation parameters, including activation energy, (E_a_), enthalpy of activation, (*ΔH*_*a*_), and entropy of activation, (*ΔS*_*a*_), for the C-steel reaction were calculated according to the Arrhenius and transition-state Eqs^78,79^. :

20 $$\:{i}_{corr}=\alpha\:{exp}^{({-{E}_{a}}/{8.314T})}$$

21 $$i_{corr}=(8.314T Nh) exp^{({\varDelta\:S_a}8.314)}exp^{({-{\varDelta\:H}_a}{8.314T\:})}$$

Where,, *h*, and N are the Arrhenius constant, Planck’s constant and Avogadro’s number.

Figure 5e illustrates the Arrhenius plot, from which the E_a_ values for C-steel, both with and without ECS treatment, can be determined based on the slope of the line (slope= -E_a_/8.314). E_a_​ represents the minimum energy required for the C-steel corrosion reaction to proceed. Table 6 shows that ECS increases the activation energy of the C-steel corrosion reaction, requiring more energy for C-steel dissolution or electrochemical reactions to occur, hindering the reaction rate. This confirms the energy barrier effect of the ECS adsorbed on the C-steel surface^52,79^. The slope and intercept of Fig. 5f represent the ΔH_a_ and ΔS_a_ values, respectively. ΔH_a_ ​ reflects the change in enthalpy (heat content) when the activated complex (ECS-Fe) is formed from the reactants. A positive ΔH_a_​ value means that the corrosion process is endothermic, requiring heat input to proceed. The presence of ECS increased the ΔH_a_ value of C-steel reaction (41.49​ kJmol^− 1^) compared to the uninhibited one (23.16 kJmol^− 1^). This further confirms that the ECS increases the energy requirement for the corrosion reaction. Both parameters are positive and higher in the presence of the prepared ECS. A positive ΔS_a_ ​ implies that the activated state is less ordered than the initial state. This usually happens when desorption of water molecules or ions from the metal surface occurs during the reaction, increasing randomness in the system. ΔS_a_ a positive ΔS_a_ ​ may indicate that the ECS promotes a reaction pathway in which water and aggressive anions are displaced by the adsorbed ECS, leading to more freedom of movement for surrounding species. These findings align with previous research^80,81^.

**Table 5:** Tafel parameters for CS in 1 M HCl in absence and presence of different concentrations of ECS at 25 ^o^ C.

	Conc.		-E_corr_,	I_corr_	βa	-β_c_	CR	*R*_*p*_	Ɵ	IE
	mM	ppm	V, vs. Ag/AgCl	µA/cm^2^	mV/dec	mV/dec	mm/y	.cm^2^.Ω		%
**1 M HCl**	00	0.00	0.456	477.56	0.123	0.129	5.549	57.38	-----	-----
ECS	4.5 x10^− 8^	25	0.477	177.22	0.085	0.098	2.059	111.65	0.6289	62.89
	9.14 × 10^−8^	50	0.452	126.4	0.094	0.113	1.468	176.01	0.7353	73.53
	1.83 × 10^− 7^	100	0.469	90.41	0.081	0.103	1.051	218.29	0.8106	81.06
	3.20 × 10^− 7^	175	0.458	48.72	0.084	0.117	0.566	437.33	0.8979	89.79

**Table 6:** Effect of temperature on the C-steel corrosion in 1 M HCl in absence and presence of 175ppm ECS.

	20 ^◦^C		30 ^◦^C		40 ^◦^C		60 ^◦^C		Thermodynamic parameters		
	I_corr,_ µA/cm^2^	IE,%	I_corr,_ µA/cm^2^	IE,%	I_corr,_ µA/cm^2^	IE,%	I_corr,_ µA/cm^2^	IE,%	E_a_	ΔH_a_	ΔS_a_
									kJmol^− 1^		J.K^− 1^mol^− 1^
**Blank**	477.56	---	880.5	---	1277.4	---	1769.8	---	25.76	23.16	20.85
**ECS**	48.727	89.79	95.395	89.16	179.72	85.93	429.54	75.72	44.09	41.49	63.64

## Surface analysis studies

### Water contact angle (WCA)

**Fig. 6:**
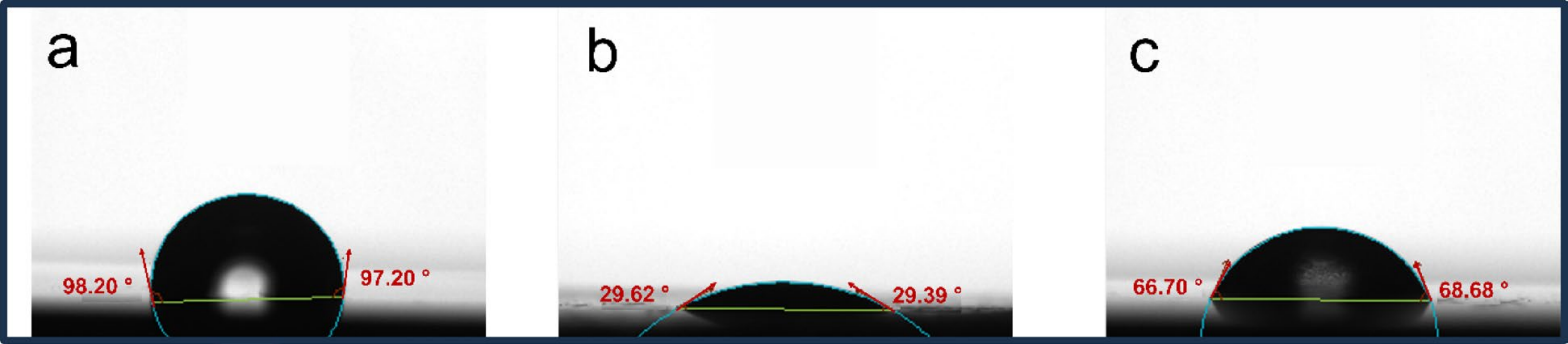
Water contact angle measurement on C-steel surface: (**a**) polished and after 24 h of immersion in 1 M HCl with (**b**) no inhibitor or/and (**c**) 175ppm of ECS.

WCA measurements provide valuable insights into the hydrophobicity of the C-steel surface and the efficacy of the ECS as a corrosion inhibitor^82^. The polished C-steel exhibited high hydrophobicity (WCA = 97.20°), attributed to a self-passivating iron oxide layer (Fig. 6a). However, immersion in 1 M HCl for 12 h drastically reduced hydrophobicity (WCA = 29.39°) due to the formation of hydrophilic corrosion products (Fig. 6b). Upon ECS treatment, WCA increased to 68.68° (Fig. 6c)., indicating the creation of a hydrophobic protective layer on the C-steel surface. The ECS’s superior performance is attributed to its molecular structure, featuring a hydrophobic alkyl chain, a quaternary nitrogen atom, and repeated polyoxyethylene units. These structural elements facilitate strong adsorption onto the C-steel surface, forming a hydrophobic barrier that effectively protects against corrosion.

### SEM and XPS analysis

**Fig. 7:**
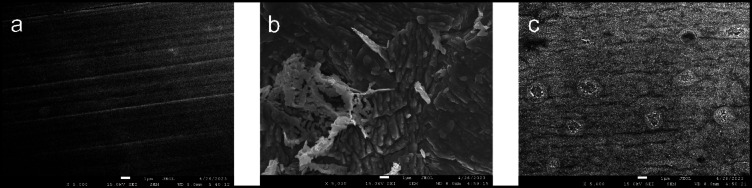
SEM images of ploshed C-steel (**a**), C-steel after immersion in 1 M HCl (**b**) and C-steel after immersion in 1MHCl + 1750 ppm ECS (**c**).

The SEM surface morphology of C-steel sheets at 5 kX magnification power, polished and immersed in 1 M HCl for 12 h, with and without 175 ppm ECS, is shown in Fig. 7a-c. The polished C-steel surface (Fig. 7a) is severely deteriorated and covered with corrosion products after 12 h of immersion in HCl (Fig. 7b). In contrast, the surface of C-steel treated with 175 ppm ECS under the same conditions appears nearly free of corrosion products (Fig. 7c). This improvement is attributed to the ECS molecules forming a protective layer that shields the C-steel surface from HCl’s offensive effects.

**Fig. 8:**
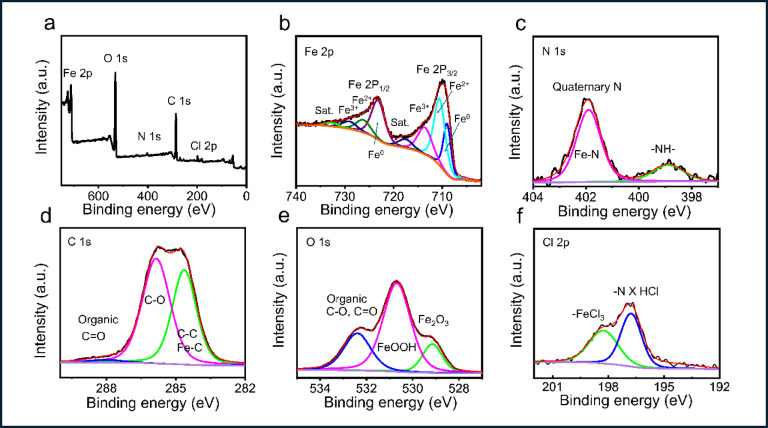
XPS survay specta (a), XPS deconvoluted profiles of (**a**) Fe 2p, (**b**) N 1*s*, (**c**) C 1 s, (**d**) O 1 s, and Cl 2p (**f**) C-steel treated with 175 ppm ECS.

The XPS survey of the C-steel surface after ECS treatment (Fig. 8a) reveals the presence of N and organic C, O atoms, and Cl confirming the formation of an adsorption layer of ECS on the C-steel surface. Figure 8b-f shows the deconvolution fitting procedure. High-resolution XPS spectrum of Fe 2p **(**Fig. 8b) depicts a double peak profile at 710.3 eV and 723.4 eV for Fe 2p_3/2_ and Fe 2p_1/2_, respectively with satellite peaks at 717.7 eV and 732.9 eV for iron oxides on the C-steel surface. The peaks at 713.7 and 729.5 eV confirm the presence of a small concentration of FeCl_3_ on the C-steel surface^83,84^. In addition to that, the peaks of O 1 s (Fig. 8e) appeared at 529.1 eV and 530.7 eV refer to the FeOH and Fe_2_O_3_^85^. Confirms the corrosion products formed from mixture of Fe-oxides and Fe-chlorides. The XPS spectrum of N 1 s (Fig. 8c) appears with peaks at 398.8 eV and 401.9 eV for the –N–H and positively charged -N^+^, respectively^85,86^. Furthermore, the peaks at 288.1 eV and 528.4 eV for C1s (Fig. 8d) and O 1 s (Fig. 8e), respectively, attributed to organic carbon and oxygen atoms, confirm the presence of ECS within the stable corrosion products on the C-steel surface. The presence of chloride may be ascribed to the acidic medium. The chloride peak Cl 2p observed (Fig. 8f) in the binding energy range between 196.8 and 198.4 eV can be attributed to N -HCl, the salt formation with the inhibitor, and FeCl_3_.

## Relationship between surface activity of prepared surfactant and corrosion Inhibition

The corrosion inhibition efficiency of the synthesized surfactant (ECS) is strongly governed by its surface activity parameters, including critical micelle concentration (CMC), adsorption free energy (ΔG°_ads_), and molecular packing behavior. The inhibitor demonstrates a preferential tendency for adsorption over micellization, as evidenced by the more negative ΔG°_ads_ compared to ΔG°_mic_, indicating thermodynamically favorable adsorption on the metal surface. The relatively low Γ_max_ suggests high surface coverage with numerous adsorbed molecules, while the elevated A_min_ value reflects a tightly packed molecular arrangement. This compact adsorption facilitates enhanced electrostatic interactions between the surfactant and metal substrate, forming a homogeneous and stable hydrophobic film that effectively blocks corrosive agents. Together, these parameters—strong adsorption affinity, optimal surface coverage, and dense molecular packing—explain the superior inhibition performance of the synthesized surfactant compared to conventional inhibitors.

## Quantum chemical study

**Fig. 9:**
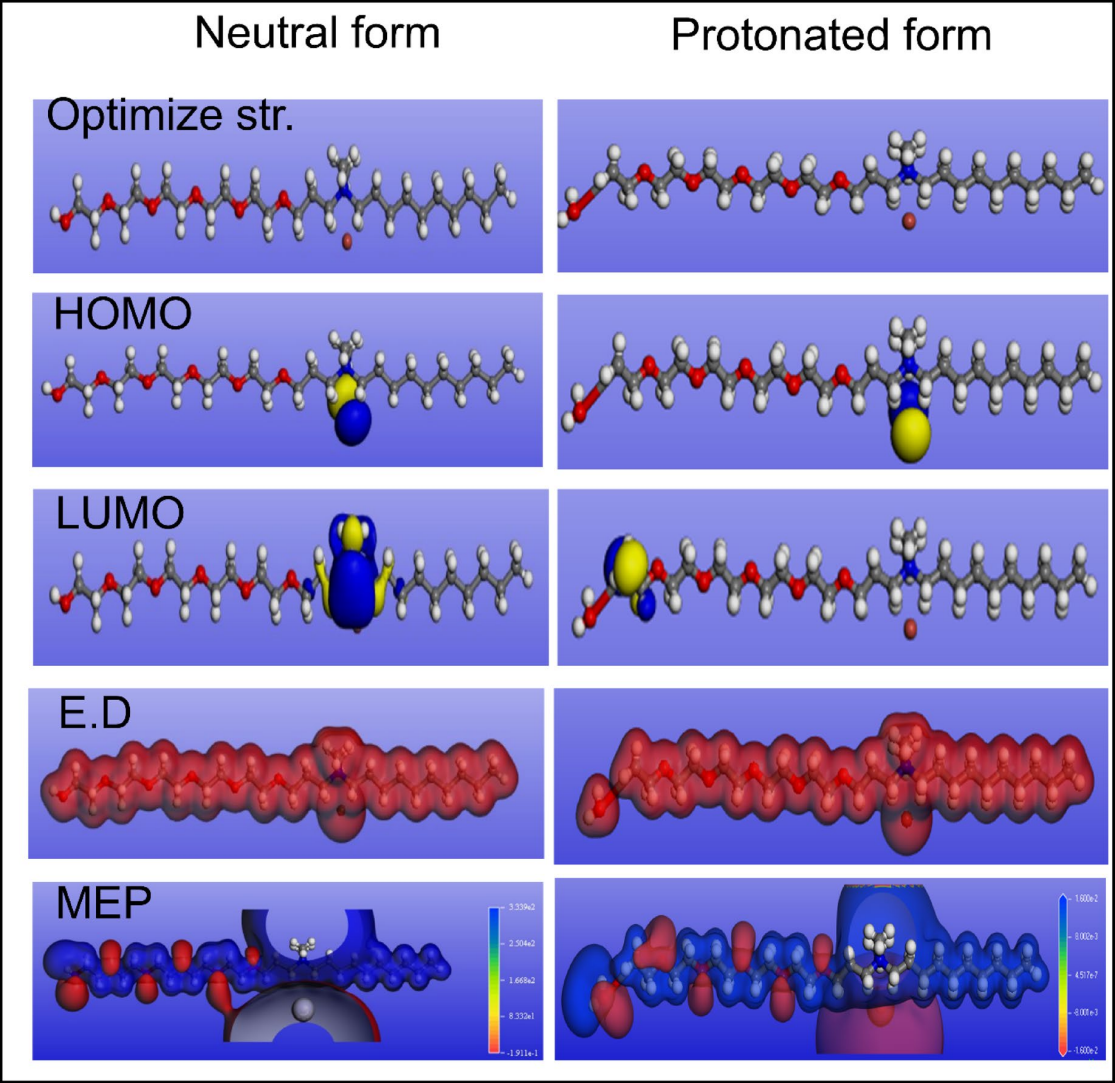
Optimized molecular structure, HOMO and LUMO and MEP of ECS molecule in neutral and protonated forms.

Quantum chemical computations save time and effort by analyzing the structure and electronic properties of synthesized organic compounds. They predict corrosion protection by assessing the chemical reactivity of organic inhibitors and elucidating the inhibition mechanism, primarily based on donor-acceptor interactions. The optimized structures of the ECS in neutral and protonated forms appeared in Fig. 9 seem to be planar which enhance the surface coverage percentage of Fe and enforce the adsorption effectiveness of the ECS’S active centers during the interaction between ECS and the C-steel substrate^18,83^. As shown in Fig. 9, in the neutral form, the electronic cloud in both the HOMO and LUMO is mainly distributed over the quaternary N^+^ and bromide atoms. In contrast, for the protonated form, the LUMO electron density is primarily localized on the terminal protonated hydroxyl group. This indicates that these regions are act as nucleophilic and electrophilic active centers in donor-acceptor interaction between the ECS and Fe^78,79^. The electron density (E.D) (Fig. 9) indicates that the ethoxylated and alkyl chain parts are charged. This synergy enhances the HOMO and LUMO activities, facilitating the formation of a compact adsorbed ECS layer on the CS surface. This occurs through electrostatic attraction and chemical bonds between the ECS and Fe. The electrostatic potential (ESP) mapping in Fig. 9 shows that the red regions correspond to nucleophilic centers, where the ECS can donate electrons to the vacant 3 d orbitals of iron. Conversely, the blue regions correspond to electrophilic centers, where the ECS accepts electrons from the filled iron orbitals via back-donation^54^. ESP mapping confirms the charge distribution at the HOMO and LUMO centers, as well as in the ethoxylated and alkyl chain parts.

According the Eqs. (1–3), the calculated energy parameters of ECS in neutral and protonated forms are listed in Table 7. Analysis of these parameters indicates the ECS adsorption capability on the Fe surface. Higher E_HO_ values enhance the ECS’s ability to donate electrons to the unfilled 3 d orbitals of Fe. Additionally, lower E_LU_ values increase the ECS’s electron acceptance affinity from the filled 3 d Fe orbitals, forming an adsorbed complex that protects the Fe surface from corrosion^87^. The energy gap (∆E) is commonly used to describe the kinetic stability and chemical reactivity of tested molecules. According to our previous work^13,26^, electron mobility is efficient when the energy difference between orbital levels is small, thereby increasing the adsorption probability. This indicates that ECS can form a stable, compact complex with Fe²⁺, creating an adhesive, smooth protective film over C-steel.

The fraction of electron transference (ΔN) displays the reactivity of ECS, is function of iron work function (4.82),$\eta_{\rm ECS}$ and **χ**_ECS_. And derived from the formula^88^:

22 $$\:\varDelta\:N=\frac{(4.82-{\chi\:}_{ECS.})}{\left[2\left({\eta\:}_{ECS.}\right)\right]}$$

According to Lukovit’s concept the positive ∆N value (2.179–4.521) indicates the affinity of ECS molecule to donate electron to form a protective complex with carbon steel^89^. The calculated parameters of ECS in the protonated form are better than those in the neutral form. This improvement may be due to the solvent enhancing the adsorption process of the inhibitor by forming hydrogen bonds, which bring the inhibitor molecules closer to the substrate surface^38^. Based on DFT calculation parameters, the experimental results have been validated by theoretical calculations.

**Table 7:** Quantum chemical parameters of the investigated ECS compound in neutral and protonated forms.

Quantum parameters	Neutral form	Protonated form
**E**_**HO**_ **(eV)**	−2.489	−3.152
**E**_**LU**_ **(eV)**	0.169	−2.195
**∆E**_**ECS**_ **(eV)**	2.658	0.957
**X**_**ECS**_ **(eV.mol**^**− 1**^**)**	1.16	2.673
**η**_**ECS**_ **(eV. mol**^**− 1**^**)**	1.329	0.478
**E**_**Back−donation**_ **(eV. mol** ^**− 1**^ **)**	0.332	0.119
$\:\varDelta\:\varvec{N}$	2.179	4.521

**Fig. 10:**
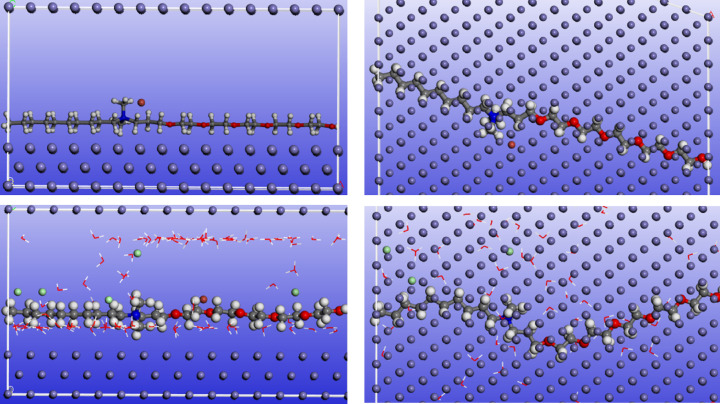
Side and top views of the adsorption mode of ECS molecule in nuetral form (upper one) and simulated acid solution (lower one) on Fe (110) substrate.

**Fig. 11:**
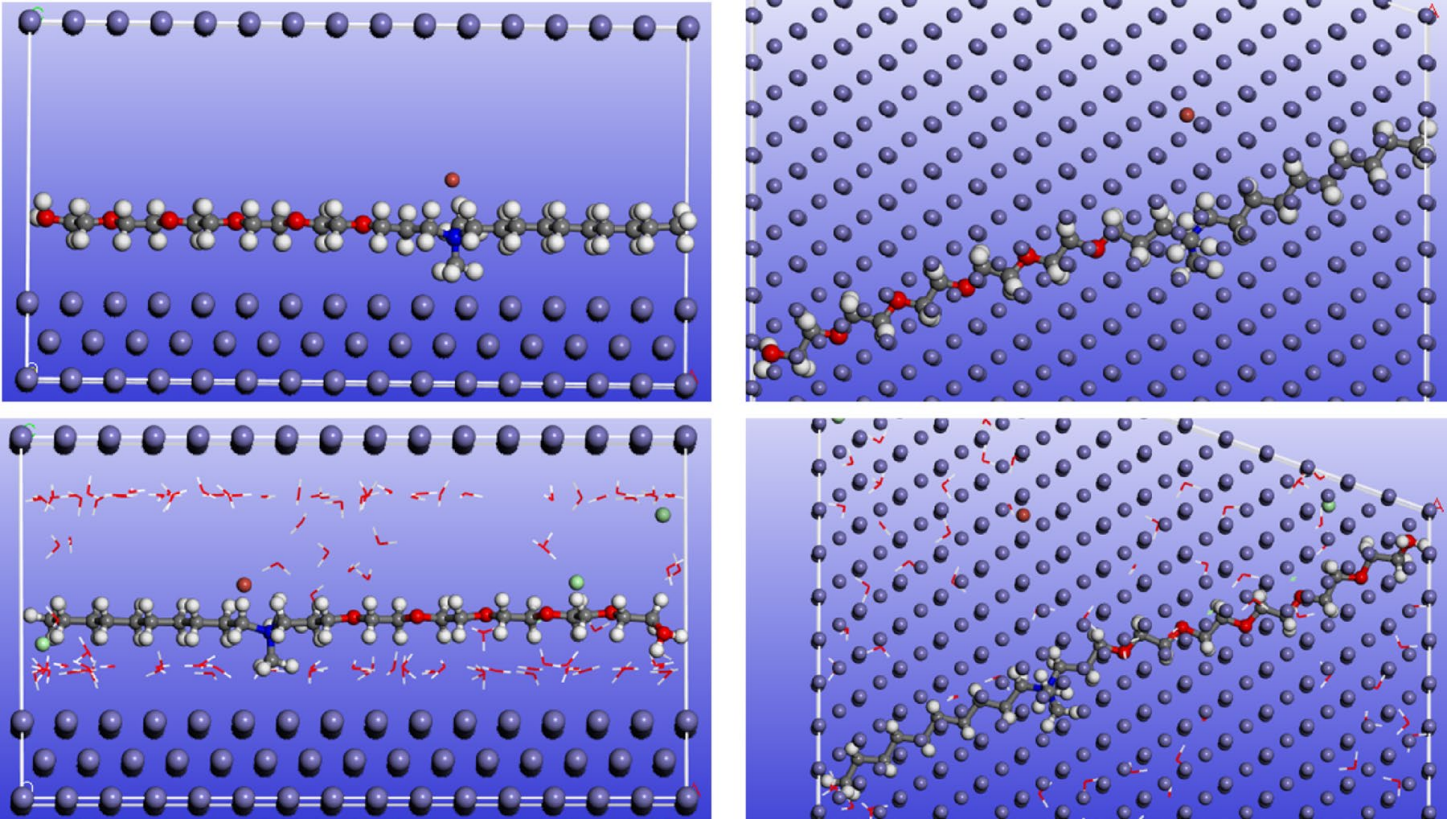
Side and top views of the adsorption mode of ECS molecule in protonated form (upper one) and simulated acid solution (lower one) on Fe (110) substrate.

The adsorption locator module implemented in M.S 6.0 Accelrys software is widely applied to mimic the real adsorption of the prospective inhibitor molecules on the C-steel surface by designing the interactions between the tested inhibitors and Fe (1 1 0) crystal by Monte Carlo Simulation (MCs) method. Figures 10 and 11 show the side and top views of the most stable geometry-optimized configurations of ECS in its neutral and protonated forms. MCs were carried out in both vacuum and simulated acidic media to closely simulate the real conditions of C-steel corrosion inhibition. The vacuum environment provides insight into the intrinsic adsorption behavior of ECS without solvent effects, while the simulated acidic solution accounts for competitive adsorption with water molecules and hydronium and chloride ions, thereby reflecting the actual electrochemical environment encountered during C-steel corrosion in HCl. The ECS molecule exhibits a nearly flat, parallel orientation to the Fe (1 1 0) substrate in both perspectives. This configuration facilitates effective interactions between the ECS and the substrate, maximizing its coverage area^40^. Table 8 presents the MCs energy parameters for the ECS/Fe (1 1 0) system. This study focuses on adsorption energy (E_ads_) as a key indicator of ECS adsorption strength on the Fe surface. E_ads_, calculated as the sum of deformation (E_def_) and rigid body (E_rigid_) energies, represents the energy released during adsorption. A more negative E_ads_ value signifies stronger adsorption^13^. The ECS exhibits strong adsorption on C-steel in both gas and simulated acid environments, as indicated by high E_ads_ values. The enhanced adsorption in the acid phase is attributed to H-bonding between water molecules and the ECS^90^. Furthermore, the E_ads_ value of ECS is higher than those of water molecules, and Cl^−^, and H_3_O^+^ ions, demonstrating its capacity to displace these corrosive species. This leads to the formation of a protective adsorption layer on the C-steel surface, safeguarding it from corrosion. All DFT and MC simulation results demonstrate that the protonated form of ECS exhibits superior adsorption performance compared to its neutral counterpart. The higher positive charge on the protonated species significantly enhances electrostatic attraction toward the negatively charged C-steel surface (due to pre-adsorbed Cl^−^ ions), while also promoting stronger interaction energies in the MC simulations. This increased affinity results in more stable adsorption, greater surface coverage, and improved inhibition efficiency. Based on DFT and MC simulations, ECS emerges as a highly promising corrosion inhibitor with exceptional performance^91,92^. The calculated ΔG_ads_ value (− 27.314 kJ·mol^− 1^) obtained from EIS data, along with the activation thermodynamic parameters (E_a_, ΔH, and ΔS), are all in good agreement with the theoretically calculated E_ads_ value.

**Table 8:** The outputs energies calculated by Monte Carlo simulation for ECS in gas and simulated acid solution phases on Fe (1 1 0).

Compound	-E_T_ (kJ/mol)	-E_ads_ (kJ/mol)	-E_rig.ads_ (kJ/mol)	-E_def_. (kJ/mol)	-(dE_ads_/dNi) (kJ/mol)			
					ECS	H_2_O	Cl^−^	H3O^+^
**ECS/Fe (1 1 0)**	346.027	295.7469	292.787	2.9590	295.746			
**ECS + 100 H**_2_ **O + 5 H3O** ^+^ **+5Cl** ^−^ **/Fe (1 1 0)**	2443.06	4441.77	2451.00	1990.77	256.72	27.16	160.08	130.19
**ECS-H** ^+^ **/Fe (1 1 0)**	333.31	30889.72	290.75	30598.97	30889.72			
**ECS-H** ^+^ **+100 H**_2_ **O + 5 H3O** ^+^ **+5Cl** ^−^ **/Fe (1 1 0)**	2487.87	35093.27	2501.52	32591.75	30965.24	25.66	164.41	131.38

## Inhibition mechanism

**Fig. 12:**
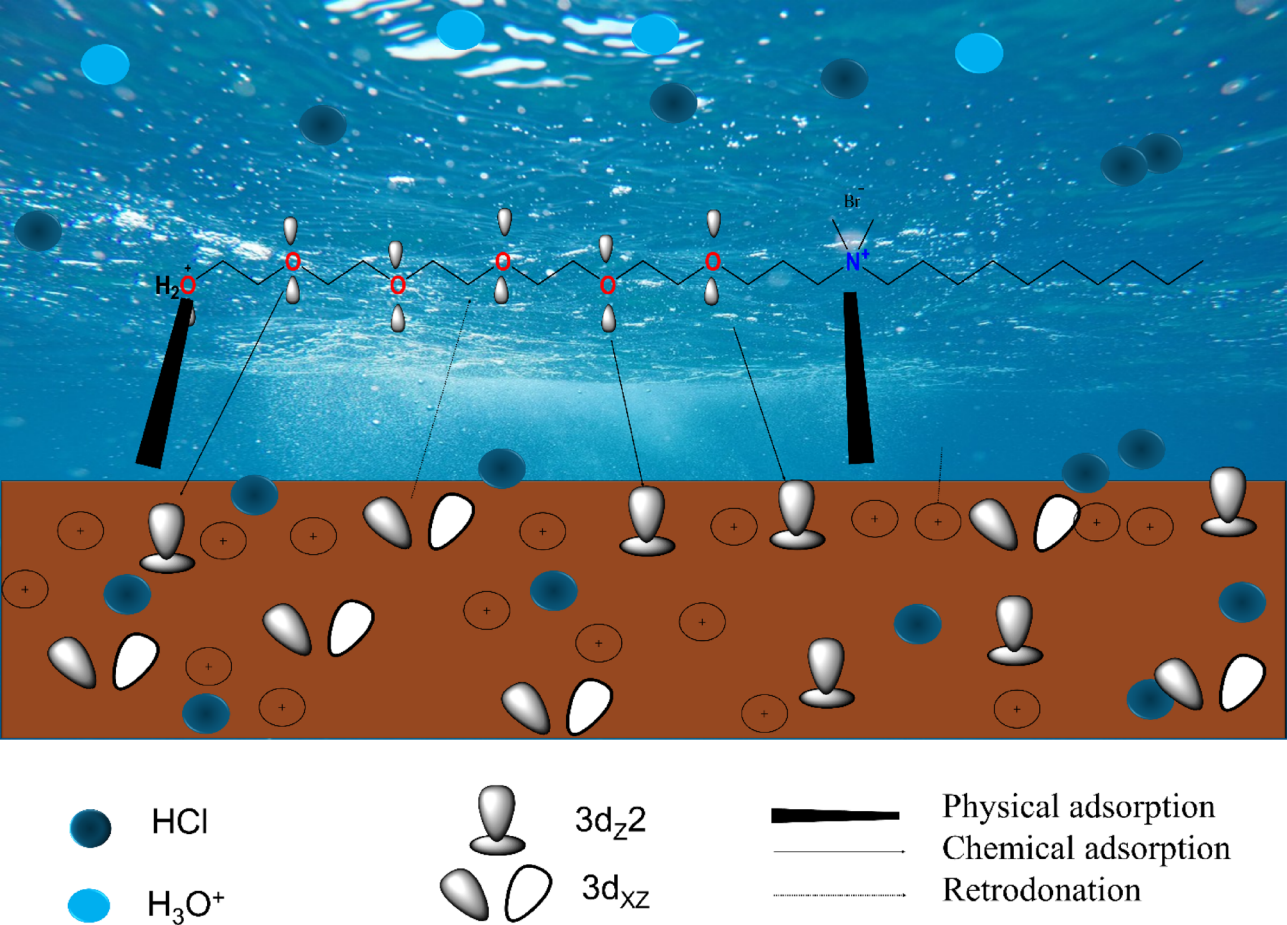
Suggested adsorption mechanism of ECS over C-steel.

ECS molecules adsorb at the C-steel/HCl interface (Fig. 12) via mixed physisorption–chemisorption (electrostatic attraction of quaternary N^+^/protonated OH groups and donor–acceptor interactions from oxygen lone pairs to Fe 3 d), with hydrophobic alkyl chain forming a compact barrier that blocks active sites, reduces charge transfer, and suppresses both anodic and cathodic reactions.

In 1 M HCl, the steel surface becomes covered with adsorbed chloride and hydronium species, which impart a negative charge to the near-surface region due to the local adsorption of Cl^−^ ions. This creates favorable sites for the electrostatic attraction of positively charged inhibitor species (ECS^+^). Protonated ECS, containing quaternary nitrogen and terminal protonated hydroxyl groups, is drawn to these negatively charged regions by Coulombic forces (Fig. 12). This rapid and reversible adsorption is consistent with the moderately negative ΔG_ads_ value (≈ −27 kJ·mol^− 1^) and the Langmuir adsorption isotherm, indicating dominant physisorption, particularly in the initial stages according to the following equations:

23 $$\:Fe\to\:{Fe}^{+2}+2{e}^{-}$$

24 $$\:{Fe}^{+2}+{Cl}^{-}\leftrightarrow\:{\left(Fe{Cl}^{-}\right)}_{ads}$$

25 $$\:{\left(Fe{Cl}^{-}\right)}_{ads}+{{H}_{3}O}^{+}\leftrightarrow\:{\left(Fe{Cl}^{-}{{H}_{3}O}^{+}\right)}_{ads}$$

26 $$\:{\left(Fe{Cl}^{-}{{H}_{3}O}^{+}\right)}_{ads}+{ECS}^{+}\leftrightarrow\:{\left(Fe{Cl}^{-}{ECS}^{+}\right)}_{ads}+{{H}_{3}O}^{+}$$

27 $$\:{\left(Fe{Cl}^{-}{ECS}^{+}\right)}_{ads}\leftrightarrow\:{\left(Fe{ECS}^{+}\right)}_{ads}+{Cl}^{-}$$

In addition to electrostatic interactions, the lone pairs on oxygen atoms within the ethylene oxide units of ECS can donate electron density into vacant Fe 3d orbitals of C-steel, forming stronger coordination bonds Also, the back-electron donation from full-field Fe 3d orbitals of C-steel to the anti-bonding molecular orbital of ECS $(\pi\ast)$ (Fig. 12). This consists the values of $\Delta N$ and E_back−donation_ calculated from DFT. This partial chemisorption further stabilizes the adsorbed layer and raises the energy barrier for metal dissolution. Monte Carlo simulations suggested that ECS molecules lie flat or nearly parallel to the Fe (111) surface, effectively covering active dissolution sites. The hydrophobic tail formed a compacted, low-permeability film that limits the ingress of corrosive species (H^+^, Cl^⁻^) and water. This orientation increases the water contact angle (WCA) and enhances the overall hydrophobicity of the C-steel surface. The calculated adsorption energy (E_ads_) for ECS is more negative than that for water, hydronium, or chloride species, indicating that ECS preferentially occupies surface sites. This displacement of solvent and aggressive ions leads to the formation of a stable, resilient protective layer.

## Comparisons with previous studies

The polyoxyethylene-based cationic surfactant (ECS) demonstrated outstanding performance as both a sulfate-reducing bacteria (SRB) inhibitor and a corrosion inhibitor for carbon steel in acidic media. The superior activity of ECS can be attributed to the synergistic combination of oxyethylene units, quaternary ammonium groups, and long-chain alkyl moieties, which collectively promote strong adsorption on the steel surface and the formation of a compact, protective barrier. As shown in Table 9, ECS achieved an inhibition efficiency of 92.23% at only 175 ppm (3.2 × 10^− 4^ M), surpassing or matching the efficiencies of several previously reported cationic surfactants such as CTAB (84.2% at 4.1 × 10^− 4^ M) and CPB (89.4% at 3.9 × 10^− 4^ M)^93^, as well as CSIII (89.3% at 5 × 10^− 3^ M)^94^. ECS also outperformed A316 (89.6% at 200 ppm)^95^ and an (E)–N-based quaternary ammonium bromide (86.44% at 1 × 10^− 2^ M)^96^, while approaching the highest efficiency reported for dodecyl polyether phthalate (94.9% at 1 × 10^− 2^ M)^97^, but at a much lower concentration. These findings confirm that ECS provides competitive, and in many cases superior, inhibition efficiency compared to both cationic and nonionic surfactants, with the added advantage of significantly lower dosage requirements, offering potential economic and environmental benefits.

**Table 9:** Comparison of the Inhibition efficiency of the ECS with the previous studies.

Name of corrosion inhibitor	Optimum conc.	IE%	References
ECS	175ppm = 3.2 × 10^− 4^ M	92.23%	Present study
Cetyltrimethylammonium bromide (CTAB)	4.1 × 10^− 4^M	84.2%	^93^
Cetylpyridinium bromide (CPB)	3.9 × 10^− 4^M	89.4%	^93^
CSIII cationic surfactant	5 × 10^− 3^M	89.3%	^94^
4,4[1,4phenylenebis(azanylylidene)bis(N, N-dimethyl-N-hexadecylaminium]bromide (A316)	200 ppm	89.6	^95^
(E) – N- (3- ((4- hydroxy – 3- methoxy benzylidene) amino) propyl) – N, N – dimethyl decane – 1- ammonium bromide	1 × 10^− 2^ M	86.44	^96^
dodecyl41-hydroxy-3,6,9,12,15,18,21,24,27,30,33,36,39-tridecaoxahentetracontyl phthalate	1 × 10^− 2^ M	94.9%	^97^

## Conclusions

FTIR and ^1^H NMR confirmed ECS chemical structure. Surface tension and conductivity measurements evaluated ECS surfactant properties in 1 M HCl. ECS exhibited strong adsorption affinity and the ability to penetrate microbial cell membranes, leading to cell death, with a microbial count of 10^4^ at 200 ppm compared to 10^6^ for the blank. Corrosion rate decreased due to ECS adsorption on C-steel, facilitated by quaternary ammonium, oxyethylene, and alkyl moieties. ECS formed a protective, high-capacitance layer, increasing R_ct_ to ~ 500 Ω.cm^2^ while decreasing the i_corr_ to 48.7µA/cm^2^ in presence of 175 ppm. PDP indicated mixed-type inhibition. Langmuir isotherm and ΔG_ads_ (−27.314 kJ/mol) suggested spontaneous physical adsorption. The SEM suggested a smooth C-steel surface after ECS treatment. The XPS spectrum of N 1 s appears with peaks at 398.8 eV and 401.9 eV for the –N–H and positively charged -N^+^, respectively. This confirmed the formation of adsorbed ESC layer over the C-steel. The ΔN parameter calculated from DFT for ECS in both neutral and protonated forms indicates the strong tendency of ECS molecules to transfer electrons to the unfilled 3 d orbitals of Fe. The adsorption energy (E_ads_) obtained from MC simulations further confirmed the adsorption of ECS and its protective role on C-steel.

## Data Availability

All data generated or analyzed during this study are included in this manuscript.
